# Higher follicular fluid glycodelin levels are negatively correlated with embryonic development in assisted reproduction

**DOI:** 10.5935/1518-0557.20180069

**Published:** 2018

**Authors:** Sibel Bulgurcuoglu-Kuran, Bilge Ozsait-Selcuk, Funda Gungor-Ugurlucan, Gözde Koksal, Can Günay, Buyru Faruk

**Affiliations:** 1 Division of Reproductive Endocrinology and Infertility, Department of Obstetrics and Gynecology, Istanbul Medical Faculty, Istanbul University, Istanbul, Turkey; 2 Department of Genetics, Aziz Sancar Institute for Experimental Medicine, Istanbul University, Istanbul, Turkey; 3 Department of Public Health, Cerrahpasa Medical Faculty, Istanbul University (Cerrahpasa), Istanbul, Turkey

**Keywords:** glycodelin, follicular fluid, embryonic development, FSH, assisted reproduction

## Abstract

**Objective:**

To investigate the possible effect of follicular fluid glycodelin levels on the quality of developing oocytes and subsequent *in vitro* embryo development.

**Methods:**

Follicular fluid glycodelin levels of 145 patients undergoing assisted reproductive treatment were analyzed and the correlation between glycodelin levels and ART outcomes were evaluated.

**Results:**

We found that glycodelin levels were negatively correlated with the number of high quality embryos on day 3 (r=-0.20, *p*=0.05). Additionally, higher glycodelin levels were correlated with higher FSH levels (r=0.18, *p*=0.04). However, glycodelin levels were not predictive for implantation (*p*=0.67) or ongoing pregnancy rates (*p*=0.99).

**Conclusion:**

Glycodelin in the follicular environment might be one of the factors that influence the competence of growing oocytes and affect the quality of subsequent *in vitro* embryo development.

## INTRODUCTION

The follicular microenvironment of the oocyte is composed of theca and granulosa cells and follicular fluid. All such components play critical roles in the development of competent oocytes and in the formation of high quality embryos (Dumesic *et al*., 2015; Sirard *et al*., 2006). The dynamics in this environment is highly orchestrated through the messengers in the follicular fluid such as miRNAs, cytokines, hormones, peptides and other molecules (Revelli *et al*., 2009). These molecules are considered as potential biomarkers for oocyte quality and assisted reproduction treatment (ART) outcomes (Dumesic *et al*., 2015; Wallace *et al*., 2012).

Glycodelin (Gd) is a glycoprotein from the lipocalin superfamily. It is synthesized in the endometrium in response to hormones progesterone and relaxin (Halttunen *et al*., 2000). Gd has three identified isoforms that share a similar protein core, but contain different glycan side chains. These Gd isoforms are expressed in different locations in the reproductive system such as the amniotic fluid (Gd A), the endometrium (Gd A), seminal plasma (Gd S), and follicular fluid (Gd F) (Seppälä *et al*., 2002). Additionally, cumulus cells are able to convert exogenous Gd A and F to Gd C, thus enhancing the zona binding capacity of spermatozoa (Chiu *et al*., 2007). Although Gd is primarily synthesized in the reproductive tract, its expression was also detected in other tissues such as the bone marrow (Kämäräinen *et al*., 1994) and gynecological tumors (Horowitz *et al*., 2001; Kämäräinen *et al*., 1996).

The importance of follicular fluid Gd levels in the timing of fertilization was previously described in a report demonstrating that Gd A and Gd F dose dependently inhibited fertilization by blocking the binding of sperm to the Zona Pellucida (Chiu *et al*., 2003; Oehninger *et al*., 1995). In contrast, Gd C favors fertilization by enhancing spermatozoa-Zona Pellucida binding (Chiu *et al*., 2007). Furthermore, it is known that *GD* mRNA is expressed in the secondary late follicles and protein Gd is taken up by the cumulus cells (Tse *et al*., 2002). The association between Gd and fertilization is well defined. However, the possible effects of follicular Gd on the competence of the developing oocyte have not been studied yet. Moreover, it is unclear whether alterations in the follicular fluid Gd levels affect subsequent embryo development.

This study aimed to investigate whether follicular fluid Gd levels have an effect on the quality of early cleavage stage embryos. Thus, we measured Gd levels in the follicular fluid of patients undergoing ART and assessed the correlation between Gd levels, embryo development and treatment outcome.

## MATERIALS AND METHOD

### Study design and patient selection

This study enrolled 176 couples undergoing assisted reproduction treatment between April 2009 and October 2012 in the Department of Obstetrics and Gynecology of the School of Medicine of Istanbul University. The inclusion criteria were: (1) presence of only one cause of infertility without additional indicators of female or male infertility; (2) absence of history of endometriosis confirmed with laparoscopic surgery; (3) absence of advanced maternal age and excess follicle stimulating hormone (FSH) levels.

Thirty-one couples were excluded from the study for not meeting the inclusion criteria. Three were diagnosed with endometriosis, two had advanced maternal age (>40 years old), five had exceedingly high FSH levels (>20mIU/ml), and 21 had undergone testicular biopsy for severe male infertility. As a result, 145 couples were included in the study. Cases of female infertility were diagnosed as ovulatory factor (n=14), tubal factor (n=18), and unexplained infertility (n=57). Fifty-six couples were diagnosed with male factor infertility only. All participants volunteered for the study and signed informed consent terms. The Ethics Committee of the School of Medicine of Istanbul University approved the study (Nº: 1228).

### Controlled ovarian hyperstimulation and assisted reproduction techniques

Controlled ovarian hyperstimulation (COH) was performed with standard gonadotropin hormone in antagonist or long agonist protocols. In antagonist protocols, recombinant FSH (Gonal F, Serono) was injected daily starting from day 3 of the menstrual cycle in combination with a fixed dose of a GnRH antagonist (Cetrorelix, Cetrotide, Serono, 0.25 mg) starting from day 6 of stimulation until human chorionic gonadotropin (hCG) administration. In long agonist protocols, a gonadotropin releasing hormone (GnRH) agonist (leuprolide acetate, Lucrin, Abbot Laboratories or triptorelin acetate, Decapeptyl, Ferring, 0.1 mg) was administered daily starting on day 21 (mid-luteal phase) of the previous cycle. After pituitary down regulation, the dose of agonist was decreased to 0.05 mg and patients received recombinant FSH (Gonal F, Serono) until human chorionic gonadotropin (hCG) administration. Ultrasound examination and serum estradiol levels were used in the assessment of follicular development. Transvaginal follicular aspiration was performed 36 hours after human chorionic gonadotropin (Pregnyl, Organon, 10,000 IU) administration.

Fertilization was performed by intracytoplasmic sperm injection (ICSI) and fertilization control was done 16-18 hours after ICSI. The quality of the resulting embryos was graded daily according to the laboratory standards modified from the ALPHA and ESHRE (European Society for Reproductive Medicine and Embryology) Guidelines (Alpha Scientists in Reproductive Medicine & ESHRE Special Interest Group of Embryology, 2011), in which Grade I (GI) included top quality embryos and Grade III (GIII) featured lower quality embryos. Embryo culture was performed in sequential mediums (G1 and G2 medium, Vitrolife) and embryos were incubated in a humidified incubator at 37 ºC with 5% CO_2_ in the air.

Elective single-embryo or double-embryo transfers were conducted on day three of fertilization under ultrasound visualization with a soft transfer catheter. The number of embryos transferred was determined based on the regulations set out by the Turkish Ministry of Health. Implantation was confirmed when a positive ß-human chorionic gonadotropin (ß-hCG) level (>10 IU/mL) was detected 12 days after embryo transfer. Pregnancy was confirmed by the verification of a fetal heartbeat on ultrasound examination.

### Sample collection

As a part of assisted reproduction treatment, follicular fluids (FF) were obtained from mature follicles measuring 18-25 mm in diameter at the day of oocyte retrieval, and were immediately transferred to sterile 15 ml conical tubes. Subsequently, FF samples were centrifuged at 400 g for 15 minutes and the supernatants were stored in liquid nitrogen for further analysis. Blood samples were drawn on day three of menstruation for hormonal profile tests including luteinizing hormone (LH), FSH, estradiol, and progesterone.

### Measurement of glycodelin levels by ELISA

Gd concentrations of the FF samples obtained from mature follicles were assessed with enzyme-linked immunosorbent assay test kits (human glycodelin ELISA kit, CUSABIO, China) with a sensitivity of 0.195 ng/ml, a detection range of 0.78-50 ng/ml, an intra-assay coefficient variation (CV) < 8% and an inter-assay CV < 10%. Samples were analyzed in duplicates and mean readings were expressed in ng/ml.

### Statistical methods

Analysis of variance (ANOVA) was used to compare between continuous variables such as age, body mass index (BMI), hormone levels on day 3 of menstruation, number of oocytes, fertilization, number of grade I and grade III embryos on day 3, number of arrested embryos, and glycodelin levels. Categorical variables such as implantation, pregnancy, and type of induction protocols were compared using the chi-square test. Correlation analysis was based on Spearman's rank correlation coefficient. *p*-values <0.05 were considered statistically significant. Values were given as means and standard deviations (SD). All statistical analyses were performed using Windows SPSS version 14.0 software (SPSS Inc., Chicago, IL, USA).

## RESULTS

### Patient characteristics and follicular fluid glycodelin levels

In the overall study group, mean female and male ages were 31.58 (±4.62; 20-40) and 34.73 (±5.31; 25-52) respectively. In order to investigate the possible association of Gd among different female infertility groups, we compared the follicular fluid Gd levels of groups diagnosed with ovulatory factor, tubal factor, and unexplained infertility. However, there were no statistically significant differences in Gd levels between different female infertility groups (ovulatory factor vs. unexplained infertility (6.91 vs. 6.53, *p*=0.79), tubal factor vs. unexplained infertility (7.82 vs. 6.53, *p*=0.26) and ovulatory factor vs. tubal factor (7.82 vs. 6.91, *p*=0.59)). Additionally, Gd levels were not different between female (ovulatory and tubal factor) and male factor groups (*p*=0.76). Table 1 presents the patient characteristics and ART outcomes categorized by cause of infertility.

**Table 1:** Patient characteristics and ART outcomes categorized according to cause of infertility.

Characteristics	Ovulatory Factor	Tubal Factor	Unexplained Infertility	Male Factor	
	n=14	n=18	n=57	n=56	*p* value
Female age (years)	33.36 (±4.73)	32.17 (±4.18)	32.19 (±4.72)	30.32 (±4.42)	0.05
Male age (years)	36.36 (±6.37)	36.94 (±5.80)	34.88 (±5.17)	33.46 (±4.77)	0.05
BMI (kg/m^2^)	24.87 (±5.81)	26.18 (±5.33)	25.79 (±4.73)	24.84 (±3.15)	0.63
FSH (mIU/mL)	8.01 (±6.17)	7.40 (±2.82)	7.51 (±3.02)	6.97 (±2.10)	0.67
LH (IU/mL)	4.74 (±4.22)	7.12 (±8.16)	5.71 (±2.92)	5.41 (±1.92)	0.33
Estradiol (pg/mL)	40.61 (±22.38)	57.66 (±30.54)	45.18 (±16.48)	47.22 (±22.23)	0.13
Progesterone (ng/mL)	0.39 (±0.19)	0.40 (±0.17)	0.48 (±0.21)	0.71 (±0.88)	0.39
Total AFC (n)	5.00 (±2.21)	6.93 (±3.37)	9.15 (±7.09)	8.60 (±4.91)	0.07
Glycodelin (ng/mL)	6.91 (±6.53)	7.82 (±4.02)	6.53 (±4.32)	7.12 (±4.06)	0.73
MII (n)	3.57(±3.59)	6.44 (±4.62)	7.42 (±5.04)	8.23 (±4.80)	0.01
Fertilization (yes,%)	85.71 (±24.15)	74.00 (±19.55)	81.67 (±16.45)	73.10 (±24.27)	0.06
GI Embryo (n)	1.33 (±1.87)	1.47 (±1.60)	2.45 (±2.91)	2.42 (±2.18)	0.36
Arrest (n)	0.57 (±0.76)	0.76 (±1.72)	1.04 (±1.69)	1.08 (±1.64)	0.69
Implantation (yes,%)	21.4	22.2	36.8	40.0	0.37
Pregnancy*(yes,%)	14.3	11.1	31.6	32.7	0.50

Values are expressed as mean ± SD. BMI, body mass index. AFC, antral follicle count. MII, metaphase II oocytes.

* Pregnancy indicates clinical pregnancy rates.

Although Gd secretion is induced by progesterone in the endometrium, no correlation was observed between these molecules in our data (*p*=0.14). However, higher Gd levels were correlated with higher D3 FSH levels (r=0.18, *p*=0.04). FSH levels were also correlated with age (r=0.18, *p*=0.04), although there was no correlation between female age and Gd levels (*p*=0.36). Additionally, there seems to be an inverse correlation between Gd levels and the number of mature follicles monitored on oocyte retrieval day (r=-0.16, *p*=0.06).

In order to test whether the ovulation induction protocol had an effect on Gd levels and ART outcomes, the outcomes of patients induced with antagonist (n=126) and long agonist (n=18) protocols were compared, although the latter group had a smaller sample size. However, no differences were seen between the antagonist and long protocol ovulation induction groups in terms of fertilization rates (*p*=0.97), implantation rates (*p*=0.66), developmental arrest up to day 3 (*p*=0.17), grade I embryo development on day 3 (*p*=0.49), and Gd concentrations (*p*=0.77) (Table 2).

**Table 2:** Patient characteristics and ART outcomes in different ovarian hyperstimulation protocol groups.

Characteristics	Antagonist Protocol	Long Agonist Protocol	
	n=126	n=18	*p* value
Female age (years)	31.79 (±4.75)	30.11 (±3.50)	0.15
Male age (years)	34.94 (±5.32)	33.33 (±5.33)	0.23
BMI (kg/m^2^)	25.45 (±4.3)	24.61 (±4.08)	0.49
FSH (mIU/mL)	7.25 (±2.94)	8.20 (±4.45)	0.25
LH (IU/mL)	5.70 (±3.96)	5.65 (±2.06)	0.96
Glycodelin (ng/mL)	7.01 (±4.50)	6.68 (±4.02)	0.77
MII (n)	6.79 (±4.63)	10.61 (±5.65)	0.002
Fertilization (%)	77.88 (±21.78)	78.11 (±17.58)	0.97
GI Embryo (n)	2.13 (±2.14)	2.70 (±2.58)	0.49
Arrest (n)	0.91 (±1.54)	1.50 (±1.97)	0.17
Implantation (yes,%)	33.6	38.9	0.66
Pregnancy* (yes,%)	27.2	27.8	0.75

Values are expressed as mean ± SD. BMI, body mass index. MII, metaphase II oocytes.

* Pregnancy indicates clinical pregnancy rates.

### *In vitro* embryo development and glycodelin levels

Gd levels were negatively correlated with the number of high quality embryos on day 3 (r=-0.20, *p*=0.05). The number of high quality embryos was negatively correlated with increasing FSH levels (r=-0.27, *p*=0.008). On the other hand, in one-way ANOVA analysis, Gd levels were not associated with implantation (implantation 6.74 (±4.36) vs. no implantation 7.07 (±4.50), *p*=0.67) or ongoing pregnancy rates (pregnancy 7.15 (±4.51) vs. non-pregnancy 7.07 (±4.49), *p*=0.99) in the study group. Additionally, there was no statistically significant difference in Gd levels between biochemical and clinical pregnancies (7.15 (±4.52) vs. 5.11 (±3.38), *p*=0.19).

## DISCUSSION

Follicular fluid is one of the main components of the microenvironment of developing oocytes. Certain molecules in follicular fluid have been previously identified as biomarkers for oocyte quality and subsequent embryo development (Dumesic *et al*., 2015, Wallace *et al*., 2012). In this study, we evaluated glycodelin levels in the follicular fluid of patients undergoing assisted reproductive treatment and observed that higher Gd levels were negatively correlated with the number of high quality embryos *in vitro*.

ICSI is one of the fertilization techniques used in ART. In this procedure, the oocyte is retrieved by follicular puncture and subsequently removed from its follicular components (follicular fluid and surrounding granulosa cells). Thus, between oocyte retrieval and fertilization by the microinjection of sperm, oocytes resting in cell culture medium are isolated from their natural environment and from the effects of follicular fluid molecules. Therefore, the impact of Gd on fertilization and embryo development in ICSI-derived embryos might reflect the influence of follicular Gd on the developing oocyte. Our finding regarding the negative correlation between Gd levels and number of high quality embryos obtained in ART cycles suggests that follicular levels of Gd might influence the development of competent oocytes. Moreover, basal FSH levels were positively correlated with FF Gd levels and negatively correlated with the number high quality embryos. These findings might suggest that with the diminishing ovarian reserve and alterations in the follicular content including Gd levels, the number of oocytes that might potentially develop into healthy embryos also diminishes. Similarly, Zak *et al*. (2010) demonstrated that Gd levels in FF from unfertilized oocytes were higher than in fertilized oocytes. The authors proposed that these results might indicate the effect of Gd on fertilization and implantation (Zak *et al*., 2010). However, they suggested that the choice of fertilization technique influenced the fertilization rate and the number of total embryos developed (fertilized oocytes on day 1), with higher Gd levels negatively correlated with IVF outcomes but not with ICSI outcomes. In our study, no correlation was seen with fertilization rates; however, Gd levels were negatively correlated with ICSI-derived good quality embryos on day 3. This conflict between studies might have arisen from the different time points (day 1 vs. day 3) at which developing embryos were assessed.

Follicular fluid is the main source of Gd F other than the fallopian tube (Chiu *et al*., 2003). In the ovary, Gd protein was first demonstrated by Immunohistochemical analysis in the stromal cells of the ovarian cortex, theca interna, and granulosa cells in the follicular phase. Additionally, Gd was detected in the theca interna of the corpus luteum and luteinized granulosa cells in the luteal phase (Kämäräinen *et al*., 1996). However, Gd levels in follicular fluid and granulosa cells were found to be lower than in amniotic fluid and decidualized endometrium (Tse *et al*., 2002). Furthermore, glycodelin mRNA expression was restricted to luteinized granulosa cells, with no expression detected in cumulus cells (Tse *et al*., 2002). No significant differences were found between the groups included in a recent study comparing follicular fluid and serum Gd levels between ART patients induced with long-GnRH agonist and antagonist protocols (Aydin *et al*., 2014). Additionally, the same authors reported in another study that FF Gd concentrations were not statistically different between clinically pregnant and non-pregnant patients, and that no significant differences were found in Gd levels between the early follicular and preovulatory phases. However, on the day of oocyte retrieval, serum Gd levels were significantly higher than in the early follicular phase (Hassa *et al*., 2015). In line with these studies, we saw no difference in Gd levels between the GnRH agonist and antagonist ovulation induction groups. Additionally, we found that Gd was not predictive for implantation or pregnancy rates in assisted reproduction. On the other hand, a study with a small sample size (Zak *et al*., 2010) demonstrated that the FF Gd levels of patients undergoing ART was significantly higher among patients with implantation failure.

Although timing of Gd secretion was investigated in serum (Bersinger *et al*., 2009), amniotic fluid (Riittinen *et al*., 1989), the endometrium (Amir *et al*., 2009; Bersinger *et al*., 2008), the fallopian tubes (Amir *et al*., 2009), and in follicular cells (Tse *et al*., 2002), there is no clear data comparing the expression levels or timing of expression of Gd subtypes throughout the female genital tract. Gd A has been the most investigated Gd type. Although undetectable around ovulation period, the highest expression of the Gd transcript was detected within the mid-luteal phase (around D2-D7) in endometrium samples on different microarray and quantitative real time PCR (qPCR) studies (Bersinger *et al*., 2008; Horcajadas *et al*., 2005; Mirkin *et al*., 2005; Riesewijk *et al*., 2003; Kao *et al*., 2002). This period overlaps with the "implantation window", a very short period of time in which embryos are permitted to successfully attach to uterine tissue. In a study conducted to detect serum levels of Gd, lowest levels were observed around ovulation whereas highest levels were detected at the end of the luteal phase, with serum glycodelin peaking around 8 days after the progesterone peak (Bersinger *et al*., 2009). Based on earlier studies reporting a correlation between higher progesterone levels and higher Gd levels during the menstrual phase (Seppälä *et al*., 1994; Joshi, 1983) and subsequent in vivo and *in vitro* studies (Taylor *et al*., 2000; Vaisse *et al*., 1990; Julkunen *et al*., 1986), endometrial Gd secretion was associated with progesterone. Several putative glucocorticoid/progesterone-responsive elements (GRE/PREs) have been identified in the gene coding protein Gd (progestagen-associated endometrial protein, *PAEP* or *GD*) (Vaisse *et al*., 1990); however, it is not clear whether progesterone is directly involved in the expression and release of Gd in the endometrium or whether Gd secretion is primarily modulated by other molecules secreted in the luteal phase such as relaxin (Seppälä *et al*., 2002; Tse *et al*., 2002; Stewart *et al*., 1997). Our study found no correlation between basal serum progesterone and FF Gd levels in ART patients. However, Gd levels were detected from luteinized follicles when sampling was performed during the oocyte retrieval procedure following 23-36 hours of hCG injection and progesterone measurements were performed on the follicular phase (day 3 of menstrual cycle). This might be a reason why we were unable to observe a correlation between progesterone and Gd levels.

A limitation of our study was FF Gd levels were not compared to serum Gd levels. Another limitation was that Gd and hormone levels were obtained at different times in follicular development.

## CONCLUSION

This study investigated the follicular effect of Gd levels on preimplantation embryo development and on the outcomes of assisted reproduction treatment. To our knowledge, this is the first report demonstrating a correlation between follicular Gd levels and quality of *in vitro* developed embryos (D3 embryos). However, the exact cellular mechanisms by which Gds impacts the development of competent oocytes and subsequent embryo development remain to be elucidated.
